# Fostering Infant/Toddler Mental Health and Language in Underserved Family Child Care Settings

**DOI:** 10.3390/children12081044

**Published:** 2025-08-08

**Authors:** Chin R. Reyes, Brooke Rumper, Reem Khamis

**Affiliations:** 1 Child Study Center, Yale University School of Medicine, New Haven, CT 06520, USA; 2 NORC at the University of Chicago, Education and Child Development, Chicago, IL 60603, USA; 3 Department of Communication Sciences and Disorders, Long Island University, Brooklyn, NY 11201, USA

**Keywords:** family child care, social and emotional learning, language development, infant, toddler, early childhood mental health consultation, mental health climate, equity, minoritized

## Abstract

**Highlights:**

**What are the main findings?**

- I-T CHILD delivered by early childhood mental health consultants to strengthen the mental health climate in family child care programs improved the quality of the language environment for infants and toddlers. In particular, there was a significant increase in child vocalizations and a significant decrease in children’s exposure to electronic media sounds.

**What is the implication of the main finding?**

- Family child care providers serving minoritized communities are capable of harnessing the power of healthy interactions with children.
- Improving the quality of the mental health climate has far-reaching effects beyond social and emotional learning; its impact on early language can serve as a protective factor for minoritized children.

**Abstract:**

**Background:** Early language development, a key predictor of later academic achievement, arises out of social interactions and communication. High-quality social and emotional interactions in early child care and education (ECCE) programs may therefore promote language-rich environments for young children. While culturally and linguistically minoritized communities face systemic barriers that limit equitable access to high-quality ECCE including social and emotional learning (SEL) programs, access to evidence-based SEL programs remains inequitable, disproportionately benefiting White, English-speaking, and higher-income ECCE providers. The current study aims to examine how I-T CHILD, a program designed to foster a climate that supports mental health and SEL in ECCE, improves the quality of the language environment using LENA technology. **Methods**: Implemented at the height of the COVID-19 pandemic, 38 family child care providers located in an urban setting (63.2% Hispanic/Latine; 40% living in poverty) were randomly assigned to the 12-week I-T CHILD program or to the waitlist-control group. Data were analyzed using hierarchical linear modeling procedures. **Results**: Infants and toddlers cared for by I-T CHILD providers produced significantly more vocalizations (*p* = 0.002; *ES* = 1.50) and were exposed to significantly less media and electronic sounds (*p* = 0.032; *ES* = −0.97) than infants and toddlers in the waitlist-control condition. **Conclusions**: Our findings reinforce the importance of the mental health climate in ECCE and its circular effect on early language development. We offer key insights into how mental health climate interventions in ECCE settings can enhance language interactions, center the child, and foster foundational skills linked to long-term academic success for historically underserved populations.

## 1. Introduction

The United States continues to grapple with the aftermath of the COVID-19 pandemic. As the country responded with physical distancing, the pandemic has also highlighted the inherent need for human connectedness. For young children, connections with caring and supportive adults are essential building blocks for resilience, cognitive development, and learning [1]. Children from birth to the age of five are exposed to trauma that can have adverse effects on brain development including the development of language. In these unprecedented times, children need healing—physically and mentally. While families struggle to accommodate the altered work schedules, financial constraints, and mental health challenges that this pandemic has brought, many children are spending up to eight hours a day in child care. The experiences of home-based child care (HBCC) programs (e.g., family child care), in particular, have not received much attention [2]. With the ramifications of the global pandemic roaring with uncertainty, the role of family childcare providers has never been more essential. The goal of the present study is to evaluate the impact of I-T CHILD, a program designed to foster a climate of mental health in family child care settings, on the quality of the language environment. 

### 1.1. Early Childhood Mental Health

Mental health is a critical determinant of economic stability and productivity both individually and collectively [3,4]. Worldwide, mental health conditions account for 9 of the 20 leading causes of morbidity and disability [4]. Moreover, over the next two decades, the global economic impact of mental health conditions is estimated to reach USD 16 trillion [5]. In the United States, the presence of mental health diagnoses in childhood will result in a lifetime loss of income of about USD 300,000 [6]. These economic costs are pre-COVID-19 pandemic estimates.

American children rank second lowest in mental health and well-being indicators compared to children from other developed nations [7], and approximately 50% of Americans will experience mental health challenges in their lifetime, most of which will originate in childhood [8]. Approximately 9% of American children receiving specialized services for mental health needs are below six years of age [9]. In early childhood, mental health conditions are often associated with externalizing behavioral problems. Children identified with severe behavioral problems at the ages three or four have a 50% chance of continuing to show difficulties throughout the elementary school years and into early adolescence [10]. Studies show that the association between these manifestations of severe mental health challenges and adolescent delinquency can begin to emerge as early as the toddler years, which makes the infant–toddler years an opportune time for early intervention and the promotion of mental well-being [11,12]. When mental health challenges emerge this young, they can negatively impact development and school readiness skills [13], and eventually, the capacity to become productive citizens. 

At a time of heightened mental health crisis during and after the pandemic [14,15], under-resourced Early Child Care and Education (ECCE) providers need to tend to their own mental health needs as well as those of the children under their care. Fostering a supportive climate of mental health in ECCE settings is one strategy to address the rising need for mental health supports in early childhood [16]. Early Childhood Mental Health Consultation (ECMHC) is an innovative approach for delivering mental health support services within ECCE settings [17]. Briefly, ECMHC has been endorsed by both the US Department of Health and Human Services and Department of Education as one of the most promising systems-level strategies for promoting early childhood mental well-being and reducing exclusionary disciplinary practices, such as expulsions and suspensions, in ECCE [18]. ECMHC is an indirect intervention where instead of taking an individual child out of their ECCE setting, a professional consultant with mental health expertise forms a collaborative partnership with ECCE staff to strengthen the climate and engage in problem-solving strategies to address behavioral challenges and other concerns [19]. Delivery models and dosage vary considerably across ECMHC services [20,21], yet evidence suggests demonstrated effectiveness [22]. Despite the recent rapid expansion of ECMHC systems across multiple states, only implementations in Connecticut [23] and Ohio [24] have been evaluated rigorously using a randomized controlled trial (RCT) design. Findings from both trials demonstrated reductions in target children’s challenging behaviors and improvements in protective factors. Notably, findings from the Ohio trial showed behavioral improvements even among randomly selected peers. To date, no ECMHC efficacy studies have focused on HBCC programs.

### 1.2. HBCC and Its Critical Service to Minoritized Communities

Children from historically minoritized communities are less likely to have access to high-quality ECCE programs and supports. Many families in these communities have limited child care options and often rely on nonformal care arrangements, which are frequently unlicensed and of unknown quality [25]. Children from minoritized communities enrolled in nonformal care may exhibit more behavioral challenges and fewer cognitive gains including language learning delays, compared to children in higher-quality child care centers [26]. Moreover, racial disparities in access to high-quality child care are most evident in nonformal settings such as HBCC programs [27]. HBCC—in the form of regulated family child care or family, friend, and neighbor care—is the non-parental child care of choice among a majority of historically marginalized families. It is especially prevalent in areas of concentrated poverty, communities of color, communities with high concentrations of people from immigrant backgrounds, and rural communities [28]. HBCC settings are often the most accessible option for families from minoritized backgrounds for several reasons. First, these settings help to address a range of structural barriers particularly for families working low-wage jobs by offering care during non-standard, unpredictable, and extended hours. Second, families often find providers within their social networks who share their home language, which can foster cultural and linguistic continuity. This dynamic also helps to mitigate fears associated with accessing public services, especially considering the growing anti-immigration sentiment in the United States [29]. HBCC providers thus offer care in ways that families value, including care that is located within their own communities, offering flexible hours, and maintaining affordability.

Approximately 1.76 million infants and toddlers in the US are cared for in HBCC settings [30]. Compared to center-based child care, HBCC has historically received less scholarly attention, and HBCC providers are less likely than their center-based counterparts to participate in or benefit from publicly funded programs and resources [31]. Furthermore, the COVID-19 pandemic exacerbated preexisting systemic issues in the child care industry, including underfunding, a limited supply of high-quality care, affordability challenges, and heightened staff layoffs and turnover [32,33]. 

Given the well-established relationship between living in under-resourced communities and early language development, which is one of the key predictors of later schooling success, this paper focuses specifically on early language development in HBCC settings.

### 1.3. Early Language Development as a Protective Factor for Minoritized Children

Early language proficiency provides the foundation for learning in formal education settings [34,35]. It is a protective factor in a child’s growth and development and is a key predictor of later development, school readiness, and schooling success [36,37,38,39]. The development of early language happens within the context of social interactions characterized by contingent reciprocity of communicative interactions found to correlate with children’s language comprehension, expression, and emergent skills [40,41]. Such social interactions are hardwired for infants and toddlers. Structural inequities—including poverty, systemic racism, and unequal access to resources—create disparities in children’s early learning environments, which can influence developmental trajectories. Research shows that infants and toddlers living in poverty who experience higher-quality early interactions perform better on standardized cognitive and language assessments [42]. However, it is important to critically recognize that these traditional measures of “development” are rooted in dominant, Western, middle-class, English-speaking norms and may not reflect the full range of culturally and linguistically diverse developmental competencies [43]. Infants and toddlers enrolled in high-quality child care centers often show faster gains on these traditional measures compared to those placed in nonformal care environments of unknown quality [26]. Higher-quality interactions in the infant–toddler years, by increasing young children’s school readiness skills, attenuate the impact of family poverty on academic achievement in later schooling [44]. This buffering effect is particularly important in educational systems that often overlook the diverse strengths of families and apply one-size-fits-all expectations to children, rather than attuning to cultural and contextual differences.

Rather than focusing on a presumed 30-million “language gap” [45], which has been widely critiqued for pathologizing families from historically marginalized communities by framing them as deficient [43], and without adequately examining how families engage with language in culturally meaningful ways or how programs are experienced by diverse communities [46], early childhood programs should prioritize affirming, identity-supportive language practices that recognize and build on families’ existing strengths. Promoting equitable access to high-quality early learning environments, particularly those that affirm children’s cultural and linguistic identities, is key to supporting children’s holistic development.

### 1.4. Early Language Development and a Supportive Climate of Mental Health in ECCE Settings

High-quality social interactions that promote language development require authentic conversations characterized by adult attunement and responsiveness to children’s communicative attempts [47,48,49,50]. A teacher who is attuned to communicative attempts understands their contribution to the curriculum with their ideas and questions, without dismissing or minimizing them. Such a teacher adjusts instructional practices accordingly, thereby creating a safe space for children to take risks. When children feel accepted and heard, they are more likely to develop language skills [48]. Conversely, if children sense that their communicative attempts will be met with disapproval or neglect, they may inhibit communication, reducing their opportunities to facilitate language and communication skills development. These types of social interactions are characteristic of climates that support the mental health of young children, in which adults provide a warm, authentic, child-centered, and empowering environment that is designed to foster children’s psychosocial well-being and holistic development, with an emphasis toward social and emotional development [51]. 

A supportive climate of mental health—or mental health climate for short—promotes social and emotional learning (SEL), which is particularly important for students who are at risk for academic difficulties [52]. SEL is “the process through which children and adults acquire and effectively apply the knowledge, attitudes and skills necessary to understand and manage emotions, set and achieve positive goals, feel and show empathy for others, establish and maintain positive relationships, and make responsible decisions” [53]. Children with well-developed SEL skills are more likely to engage meaningfully in the learning process, perform well academically [54,55], and grow into responsible citizens in adulthood [56]. It is therefore not surprising that SEL skills have been found to be necessary indicators of workforce success, including employment status, job performance, wages, and entrepreneurial success [57]. 

Teachers who integrate SEL into their classroom practices can support more positive educational experiences for children. A meta-analysis involving over 270,000 children from preschool through high school found that children who participated in SEL-based programs demonstrated achievement gains of 11 percentile points compared to those who did not [58]. Fostering SEL is particularly critical during early childhood, as young children’s SEL skills in this period have been shown to predict academic achievement in elementary school [59,60] and are closely associated with gains in language proficiency [61]. Language provides a key mechanism through which children learn to label, articulate, and express emotions; developing these emotional competencies, in turn, requires foundational language processing skills [62]. Taken together, these findings suggest that SEL and language development scaffold one another, forming an interconnected foundation for later learning and social success.

Investigations of the interrelationship between SEL and language have primarily focused on the quality and quantity of parents’ verbal interactions with their child (“child talk” or “language input”), with limited attention paid to children’s language engagements in non-home contexts, such as in ECCE settings. Most of these studies examined conversational turn-taking as a critical component of social interactions that supports children’s SEL development. Fewer studies, however, have examined the broader climate of emotionally supportive interactions as a context for learning and development. 

Existing evidence suggests that parent–child conversational turns at 18 months contribute significantly to the development of attachment, emotion regulation, and emotion communication competencies at age 30 months [63] and predict later emotional regulation at 77 months, particularly when children are presented with a conflict with parent protocol [64]. Conversational turns at 30 months were also found to correlate with social and emotional cognition, regulation, and emotional communication at 77 months. These findings suggest a potential causal relationship between the use of conversational turns in parent–child interactions and the development of SEL skills, with implications that extend into the school-age years as well [63]. 

While these findings offer valuable insight for parent education and for understanding the interplay between child talk and SEL, such studies warrant critical examination through an equity lens. In particular, they may inadvertently obscure the role of broader social and institutional factors, practices, and policies that shape children’s developmental outcomes [65]. Shifting the responsibility for educational disparities onto parents—without acknowledging the institutional systems that produce and sustain inequities—reinforces deficit-based ideological conceptualizations of imbalances in final achievements such as the “achievement gap” discourse [66,67,68,69]. Moreover, the standardization and universality of many parent education programs—often rooted in quantitative assessment tools—can reflect a deficit ideology that upholds white supremacist norms and marginalizes alternative cultural knowledge systems [43,68,70]. 

From a social justice-oriented perspective, fostering the mental health climate within HBCC settings may be a more inclusive and systemic approach to advancing SEL. Such efforts not only support the social and emotional development of children which been associated with language and academic gains but also promote the transformation of ECCE services at a larger scale into evidence-based, culturally responsive systems [71]. Equitably reframing the conversation requires a shift away from emphasizing parental responsibility toward a greater focus on institutional environments. For example, a recent study found that children’s social and emotional development is mediated by teachers’ perception of their workplace climate [72]. In this study involving 329 children and their 53 teachers across 13 ECCE settings—including Head Start programs—children’s SEL outcomes were positively associated with a teachers’ sense of community. Specifically, greater teacher collegiality and engagement reports were associated with lower levels of aggression and anxiety among children. 

Hence, there is a need for a more dynamic and non-linear understanding of the relationship between language use and SEL, one that includes an examination of the mental health climate within ECCE settings. One of the main debates in this domain relates to the bidirectional relationship between language use and SEL; however, there are limited empirical studies addressing this question [64]. Current understandings are largely based on examinations of language use, typically measured through indicators of parent talk at home (e.g., adult word counts and conversational turns), and SEL outcomes, typically measured by children’s competencies at the individual level) in longitudinal studies [64] or in cross-sectional studies, through a combination of researcher-administered SEL assessment and teachers’ reported classroom adjustment and academic readiness [73]. These investigations would benefit from an expanded focus to also include interactions occurring in ECCE settings. There is limited empirical evidence exploring the mental health climate of ECCE settings and its effect on children’s SEL development, particularly in early childhood; most existing research focuses on school-age children [74,75]. This is despite of a broader body of evidence linking the overall quality of ECCE settings to children’s social and academic achievement, and a growing recognition of the need to evaluate not only structural features of ECCE quality but also process-based features such as interaction quality and emotional climate [76]. There is also value in broadening the focus from individual children’s intrinsic performances to a more ecological perspective that considers the relational climate in which children are embedded, along with the multitude of experiences affecting social and emotional development when considering the linguistic, sociocultural, and historical contexts of these experiences for different groups of children. This broader lens is critical for informing public policy, as recommendations derived from parent-focused investigations risk overlooking institutional level dynamics and reinforcing deficit-based narratives. For example, while acknowledging that the field is still in its “embryonic state”, Gómez Muzzio (2022) discusses public policies aimed at encouraging parents to talk more with their babies as a key strategy for promoting SEL development across the life span [64]. However, a sole focus on home-based conversational interactions may obscure the role of non-parental care environments, especially those serving working-class and racialized communities, in shaping SEL trajectories. Without adequate attention to these broader settings, policy recommendations may unintentionally reinforce structural inequities by shifting responsibility to individual families while neglecting systemic reform.

### 1.5. I-T CHILD

The Infant–Toddler Climate of Healthy Interactions for Learning and Development (I-T CHILD) Tool [51] is designed to integrate seamlessly with extant service delivery mechanisms such as ECMHC. Briefly, I-T CHILD serves both as an assessment tool and a consultation framework. As an assessment tool, scores on I-T CHILD measure the current climate of the program being observed. These include the following:

1. *Transitions*—Smooth, efficient, flexible, and productive transitions between activities;
2. *Directions and Rules*—Behavior management characterized by setting, modeling, and enforcing clear, consistent, and developmentally appropriate rules of conduct and applying proactive and positive behavior strategies.
3. *Social and Emotional Learning*—Fostering emotional literacy, relationship skill-building, and social problem-solving;
4. *Adult Awareness*—Monitoring and attunement to both overt and subtle signals and signs for assistance;
5. *Adult Affect*—Emotional state of adults;
6. *Adult Cooperation*—Adults’ demonstration of teamwork, camaraderie, and genuine enjoyment of each other’s presence;
7. *Adult–Child Interactions*—Adult interactions with children characterized by dignity, respect, genuine relationships, equity, and the celebration of diversity;
8. *Individualized and Developmentally Appropriate Practices*—Promotion of holistic development through a child-centered and individualized approach;
9. *Child Behaviors*—Child behaviors exhibiting positive affect and self-regulation.

Scores on I-T CHILD then inform areas of effectiveness that promote a supportive mental health and identify opportunities for reflection and growth. As a consultation framework, I-T CHILD-certified consultants implement the *Consultant’s Guide to the CHILD* [77] to frame and guide the process with the provider. I-T CHILD reframes traditional paradigms by shifting: (a) from focusing on the child to supporting the adult; (b) from centering on pathology to spotlighting pedagogy, elevating effective practices, successes, and learning; (c) from resorting to child pull-out to emphasizing adult attunement. 

I-T CHILD tool, originally developed for center-based ECCE programs, was refined to address the context of HBCC [51]. I-T CHILD was pilot-tested as a systematic and structured framework for ECMHC in HBCC programs. 

### 1.6. The Present Study

The present study aims to evaluate I-T CHILD as a framework for delivering ECMHC to HBCC programs (specifically, family child care) serving infants and toddlers in a culturally and linguistically minoritized urban community. In particular, this study focuses on the impact of the I-T CHILD framework on early language development. This is the first RCT of ECMHC delivered to family child care providers in a linguistically diverse setting, and the first RCT of ECMHC implemented during the COVID-19 pandemic. 

We hypothesize that providers coached to use the I-T CHILD framework will create richer language environments and that infants and toddlers in these settings will demonstrate more advanced language use than providers/infants and toddlers in the waitlist-control group.

## 2. Methods

### 2.1. Procedures

The COVID-19 pandemic placed unprecedented demands on systems that historically relied on face-to-face communication, including reading and responding to non-verbal and paraverbal cues. The implementation of creative strategies was warranted to adjust to the “new normal”. Before running the full RCT, we conducted a pilot study in the fall of 2020, during the height of the pandemic, with support from local community-based organizations. The lead organization’s Internal Institutional Review Board (IRB) approved both the pilot (IRB #2000026231) and RCT (IRB #2000030742). 

### 2.2. Pilot Study

We conducted a virtual “Meet-and-Greet” event, held in the evening, which was attended by over 50 family child care providers. The session was facilitated in both English and Spanish. At the end of the recruitment period, 14 providers enrolled and participated in consultation. I-T CHILD-certified ECMHC consultants from local community-based organizations conducted virtual observations and one-on-one sessions with providers, which lasted for 3–4 months (16–24 h total).

We collected I-T CHILD scores based on consultants’ observations and providers’ surveys. Despite translating and back-translating the surveys, response rates from providers were low. Most providers did not respond to email reminders or declined offers to complete surveys over the phone with a research team member. Consultants noted that many providers, particularly those with limited literacy, expressed difficulty in understanding the survey questions despite our efforts.

The pilot study informed several procedural refinements that we addressed in the full RCT through the following: (1) expanding our modes of communication (e.g., e-mail, phone calls, text alerts) to improve response rates; (2) ensuring linguistic and cultural matching between consultants and providers to increase rapport and engagement; (3) incorporating a more objective outcome measure Language Environment Analysis (LENA) technology (lena.org) to address issues of social desirability and provide more objective measures. The LENA system includes software and a wearable “talk pedometer” that collects up to 16 h of continuous speech data in a child’s environment as a reliable measurement of the quantity and quality of the spoken language the child is exposed to in the family child care environment. 

### 2.3. The Current Study (RCT)

Family child care providers were recruited through child care resource and referral agencies and community partners in a multilingual, multicultural urban city in the Northeastern United States. Similar to the pilot study, we conducted a virtual “Meet-and-Greet” session to build rapport, foster trust, and address any questions participants had about the study. 

From December 2020 to March 2021, providers were invited to complete an eligibility survey. To participate in the RCT, providers were required to fulfil the following: (a) be a licensed home-based child care provider serving at least one infant or toddler; (b) be located within the study-funded catchment area; (c) agree to receive the I-T CHILD intervention as part of ECMHC; (d) consent to participate in data collection. Providers who did not meet all inclusion criteria were excluded from the study. Once deemed eligible, providers submitted a list with all the children under three who were enrolled in their care, using the child’s initials and date of birth as identifiers. A maximum of four children were sampled per provider. Rosters of the children 36 months or younger enrolled in the project were obtained from the providers. If more than four children 36 months or younger sent in consent forms, four children were randomly selected.

Providers completed questionnaires and agreed to allow research assistants to visit their programs to install the data collection software for data collection (see Section 2.5). Providers received up to USD 400 each for completing all waves of data collection. 

Providers were randomly assigned to either the intervention group (I-T CHILD) or the waitlist-control group. Random assignment was conducted by simple (unweighted, unstratified) randomization using an online random number generator (random.org) once all the providers completed the needed documentation to participate in the study. I-T CHILD was delivered by existing ECMHC consultants affiliated with local child care resource and referral agencies and community-based organizations. ECMHC consultants were licensed or license-eligible mental health providers with additional training in providing consultation to ECCE programs for children younger than kindergarten entry age. 

Consultants participated in a four-day virtual training. At the end of training, they were required to pass reliability before they were able to begin consulting with HBCC providers. Reliability was established by having consultants score several videos of early childhood classrooms using the I-T CHILD tool and achieving at least 80% alignment with master codes (defined as either scoring within the “master score” or the “acceptable score”). Additionally, consultants participated in a four-day, three-hour facilitated training focused on implementing I-T CHILD.

The intervention consisted of approximately 10 one-hour consultation sessions conducted over 10–12 weeks at each provider’s setting. On average, providers completed 15.41 h of consultation (*SD* = 7.46) across 8.63 sessions (*SD* = 2.12) spanning over 11.72 weeks (*SD* = 3.83). While most sessions occurred in person (*M* = 6.34, *SD* = 3.11), some were conducted remotely (*M* = 2.26, *SD* = 2.78) due to extenuating circumstances (e.g., consultant pregnancy, COVID-19 precautions). 

During the first session, consultants conducted a pretest I-T CHILD observation. The scores from this initial observation were used to identify areas of strength and growth for each provider, and to tailor subsequent consultation sessions to align with each provider’s individual needs. Upon completion of the final consultation session (12-week period), consultants conducted a posttest CHILD observation. 

Consultants had an average of two years of experience as an ECMHC consultant (*SD* = 1.29). Six of the nine consultants had prior experience as an early childhood educator (*M* = 8.20 years of working in early childhood education; *SD* = 7.29). Most consultants held a master’s degree or a professional degree in psychology, social work, or a related field (*n* = 6). All consultants identified as females and their racial/ethnic identities included Hispanic/Latine (*n* = 4), Asian (*n* = 3), and Black or African American (*n* = 2). Nearly half of the consultants (44.40%) reported speaking Spanish very well and 22.20%. reported speaking Chinese well or very well. Notably, every effort was made to ensure linguistic alignment between consultants and providers, and providers were able to receive consultation in their preferred language. Consultation was conducted in English, Spanish, and Chinese.

### 2.4. Participants

A total of 50 family child care providers completed consent forms. Twelve providers withdrew from the study before completing the intervention due to various reasons (e.g., insufficient time to participate fully or loss of interest in data collection amid the stress of the COVID-19 pandemic). 

The final sample consisted of 38 family child care providers. The majority of the providers identified as Hispanic/Latine (63.2%), followed by Asian (18.4%), Black/African American (15.8%), and White/Non-Hispanic/Latine (2.6%). Regarding preferred language, 50% indicated Spanish, 34.2% English, 5% Chinese, and 2.6% Polish. Most providers had earned their Child Development Associate (CDA) credential (47.4%), while 23.7% had completed up to high school or earned a GED. The remaining participants held a bachelor’s degree (15.8%), associate’s degree (7.9%), or master’s degree (5.3%). 

Regarding public assistance, 14 providers (36.8%) received Medicaid, two providers (5.3%) received Supplemental Nutrition Assistance Program (SNAP), and one provider (2.6%) received Women, Infants, and Children Nutrition Program (WIC). No providers reported receiving Temporary Assistance for Needy Families (TANF). Approximately 40% of providers fell below the federal poverty threshold, with providers in the I-T CHILD group being compared to the waitlist-control group [73.33% vs. 26.67%, χ^2^(1) = 4.26, *p* = 0.039)]. 

The primary motivation for choosing the profession for 55.3% of providers was the desire to apply knowledge in child development or to develop skills in caring for and educating children. Additional motivations included wanting to help children and families (23.7%), the convenience of operating a child care business while raising their own children (13.2%), and financial reasons (5.3%). One provider (2.6%) mentioned that loving children was her primary motivation for being a family child care provider. On average, programs had been operating for 11.68 years (*SD* = 9.33). Across the 38 participating programs, 332 children were enrolled. Among them, 44.28% were under 3 years old, 40.06% were between 3 and 5, and 15.66% were 6 years or older. Most children in the study (62.6%) were learning a language other than English at home. Reported home languages included English (44.28%), Spanish (28.01%), Chinese (11.45%), French (2.71%), Korean (0.90%), Arabic (0.60%), Bengali (0.60%), and Haitian Creole (0.30%). Additionally, 11.14% of children were multilingual. 

To quantify racial and linguistic diversity, we calculated Simpson’s formula [78], with scores ranging from *0* (low proportions and representations in race and languages) to *1* (high proportions and representations in race and languages). The mean diversity index was 0.16 (*SD* = 0.17) for racial diversity and 0.29 (*SD* = 0.23) for linguistic diversity. Children in participating programs were funded by a variety of sources, including parent pay, federal/state funding, and a combination of federal/state/parental funding streams. 

The present study draws on a subset of participants who completed LENA data collection at both pretest and posttest (12 weeks after the intervention). Due to pandemic-related concerns (e.g., reluctance to allow research assistants into the home) and logistical challenges (e.g., limited equipment, family mobility, and child attendance), the final analytic sample included 28 providers (32% living below the federal poverty threshold; 57% receiving services to be delivered in non-English language) and 72 children (35 in the waitlist-control, 37 in the I-T-CHILD group). Among these children, 55.6% were female, and ages ranged from 5 to 33 months [*M* = 21.50; *SD* = 7.94]).

### 2.5. Measures

#### 2.5.1. CHILD for HBCC Observation Tool

Consultants rated the providers they were serving both pretest and posttest using the *Climate of Healthy Interactions for Learning and Development for Home-Based Child Care Observation Tool* (CHILD-HBCC) [51]. CHILD-HBCC is an observational measure designed to assess the quality of home-based child care environments for infants and toddlers. It was adapted from the center-based child care version developed for preschool settings. This measure is completed by a trained and certified observer during an approximately two-hour observational period; in this study given the pandemic, observation was limited to one hour (see Reyes & Gilliam for a similar protocol) [24]. The CHILD for HBCC tool consists of 28 easily observable items along nine dimensions (described earlier). Items reflect common behaviors and interactions that occur in early childhood education settings. Each item is scored using a five-point anchored Likert scale ranging from −*2* (very/consistently undermining children’s learning and development) to +*2* (very/consistently promoting children’s learning and development). A score of *0* represents a baseline expectation, indicating interactions that are neither undermining nor promoting. Table 1 shows the progression from baseline expectation to promoting levels following the principles of mentally healthy climates [51]. When the principles are applied to a particular item in a dimension, such as the case of the item, emotion-oriented interactions under the SEL dimension, the rubric for the item ranges from −*2* (dismissive of feelings) to +*2* (honoring feelings), with a score of *0* reflecting perfunctory acknowledgment of feelings. Each item across all the dimensions have specific scoring rubrics.

#### 2.5.2. Language Environment Analysis (LENA)

LENA technology is a validated, automated system used to measure the quantity and quality of language input received by infants and toddlers in naturalistic settings [79]. LENA has been used in over 100 peer-reviewed publications across diverse racial and ethnic populations, and across research and clinical domains, including studies with typically developing children and those with developmental language disorders, hearing impairments, and autism spectrum disorder [80,81,82]. Its widespread use stems from its ability to produce ecologically valid, automated data on language input and output over extended periods. In this study, the LENA system was used to capture and analyze the language environment of children enrolled in family child care programs. The system includes a lightweight digital language processor (DLP), often referred to as a “talk pedometer” that is worn by the child in a specially designed vest. The device passively records all auditory input within hearing range of the child for up to 16 h. The LENA device is collected and connected to a computer with LENA software installed. The LENA software processes the audio recordings into quantitative metrics such as adult word count, child vocalizations, conversational turns, and background electronic noise, providing an estimate of the child’s language environment and verbal productions. 

In this study, trained research assistants conducted all LENA-related data collection in person, following a standardized protocol for observational and technological procedures. LENA data collection was administered at pretest and posttest timepoints for providers in both the intervention and waitlist control groups. 

The LENA protocol included detailed guidelines for pre-visit responsibilities, communication, day of visit tasks, and post-visit steps. Prior to each visit, research staff confirmed that participating sites had the necessary material: fully charged LENA devices, functional LENA vests, LENA USB cables, and access to a computer with LENA Hub installed. On the day of the visit, research assistants verified child assent to wear the vest and ensured the device was securely and comfortably placed inside the garment. If children were not wearing their vest upon arrival, assistants checked the battery status and assisted reinitiating the wearing process as needed. 

The data collection protocol served as a checklist to ensure procedural consistency, fidelity, and cultural responsiveness in data collection, with particular emphasis on relational approaches. All procedures adhered to confidentiality protocols and respectful communication, multilingual coordination with providers, and flexible scheduling to minimize disruption—especially in light of the added challenges of the COVID-19 pandemic. In a second visit, after the recording period concluded, research assistants uploaded the audio data and confirmed successful transfer using secure LENA Hub version 4.1 software and charging of devices for future use. 

Importantly, data collectors were blinded to group assignment (I-T CHILD vs. waitlist-control) to reduce the risk of bias in data collection. Oversight was provided by a project coordinator with dual expertise in infant mental health and speech-language therapy. The coordinator was responsible for training research assistants, supervising field operations, addressing any unexpected issues, and maintaining weekly communication with the larger research team. This role ensured continuity and quality across all phases of data collection and supported adherence to IRB protocols and project timelines.

Once collected data is uploaded to a computer using the LENA Hub software, the software automatically segmented and quantified specific linguistic features without the need for manual transcription or annotation [83]. The primary LENA outcome metrics included the following:

1. *Adult Word Count (AWC)*: Total number of adult words spoken in proximity to the child wearing the device.
2. *Child Vocalizations (CV)*: Number of speech-like utterances produced by the child wearing the device.
3. *Conversational Turns (CT)*: Number of alternations between adult and key child within five second window.
4. *Electronic Media Exposure (TV/Electronic)*: Duration of exposure to television or other electronic sounds (non-speech categories).
5. *Meaningful Speech*: Speech that is both clear and proximal to the child.

To ensure comparability analysis across recordings of varying lengths, measures of AWC, CV, and CT were converted to rates per hour, and TV/electronic exposure and meaningful speech were converted to seconds or percentages of total time. These standardized metrics constituted the primary outcome variables for the current study.

### 2.6. Analytic Strategy

The primary analyses focused on the effect of the I-T CHILD intervention on early language environments, as measured by LENA metrics. The main analysis employed hierarchical linear modeling (HLM) procedures [84], which was deemed the appropriate analytic approach as it can reflect the nested structure of the data (children nested in family child care programs) to avoid overestimation of significance. This approach deals with unmeasured variability at different levels by allowing the residuals to be partitioned at each level. Our analyses treated child-level variables as Level 1 and FCC provider-level characteristics as Level 2. Data were estimated in HLM 8 [85], using restricted maximum likelihood estimation with robust standard errors, applying grand mean centering for interval-scale variables or uncentered analysis for dummy-coded variables. Prior to conducting the primary analyses, we ran a null (unconditional random intercept) model to calculate intraclass correlation coefficients (ICCs) in order to assess the proportion of variance attributable to program-level clustering. The calculated ICCs exceeded 0.50, indicating a substantial proportion of variance at the program level and confirming that HLM was warranted.

Next, we assessed baseline equivalence of LENA pretest scores between I-T CHILD and waitlist-control groups following What Works Clearinghouse guidelines (https://ies.ed.gov/ncee/wwc/handbooks, accessed on 21 July 2025). On two measures, baseline equivalence was not achieved (Hedges *g* [ES] > 0.25; see Table 2). Although no significant differences between the I-T CHILD and waitlist-control groups were found (ps > 0.10), effect sizes exceeded the threshold for adult word count (*ES* = −0.52) and meaningful utterances (*ES* = −0.47), both favoring the waitlist-control group as scoring higher than the intervention group. We then ran exploratory analyses to determine which covariates to include in the model. Although none of the demographic variables were consistently associated with the LENA scores, we added the poverty status of the family child care provider (*1* = household income falls within poverty threshold) given the strong link between language and living in poverty [86,87]. We also added the pretest scores as covariates regardless of baseline equivalence to standardize analyses. The final model is represented as

Posttest LENA*_ij_* = β_0*j*_ + β_1*j*_(pretest LENA) + r*_ij_*

β_0*j*_ = γ_00_ + γ_01_(I-T CHILD*_j_*) + γ_02_(Poverty*_j_*) + *u*_0*j*_

β_1*j*_ = γ_10_

In this model, the posttest LENA score for child*_i_* in program*_j_* is modeled as a function of the child’s pretest LENA score, as well as of Level 2 characteristics that include treatment status (*1* = I-T CHILD) and provider poverty status (*1* = below poverty rate). Effect sizes (ESs) were calculated using Hedges *g* to account for small sample size bias in standardized mean differences.

## 3. Results

### 3.1. Mental Health Climate

Consultants rated the mental health climate (I-T CHILD) in the providers’ homes prior to and after consultation. As shown in Table 2, consultant ratings improved posttest across most dimensions of CHILD-HBCC except for adult awareness (trend only), adult affect, and child behaviors. Note that these three dimensions were already rated high at pretest (approaching +1 score), indicating these behaviors were already aligned with the promoting range of the scoring rubric. 

Overall, improvements across the remaining dimensions indicate that (1) transitions between activities (which often are the most challenging times for young children) became smoother and more developmentally responsive to children’s needs; (2) challenging behaviors were scaffolded more effectively with clearer behavioral expectations and the use of proactive, developmentally appropriate strategies; (3) providers were more attuned to children’s emotions and fostered more collaborative relationships with peers; (4) staff members demonstrated greater teamwork and collaboration; (5) providers exhibited more equitable treatment of children; (6) there were more child-centered practices with increased individualized support. A particularly notable finding was the improvement in the SEL dimension, which demonstrates a marked shift from undermining behaviors, denotated by a negative score, to a baseline expectation score of zero. This suggests a change in providers’ behavior from dismissal/no acknowledgment of children’s emotions to consistently acknowledging feelings. 

### 3.2. Early Language Development 

**Figure 1:**
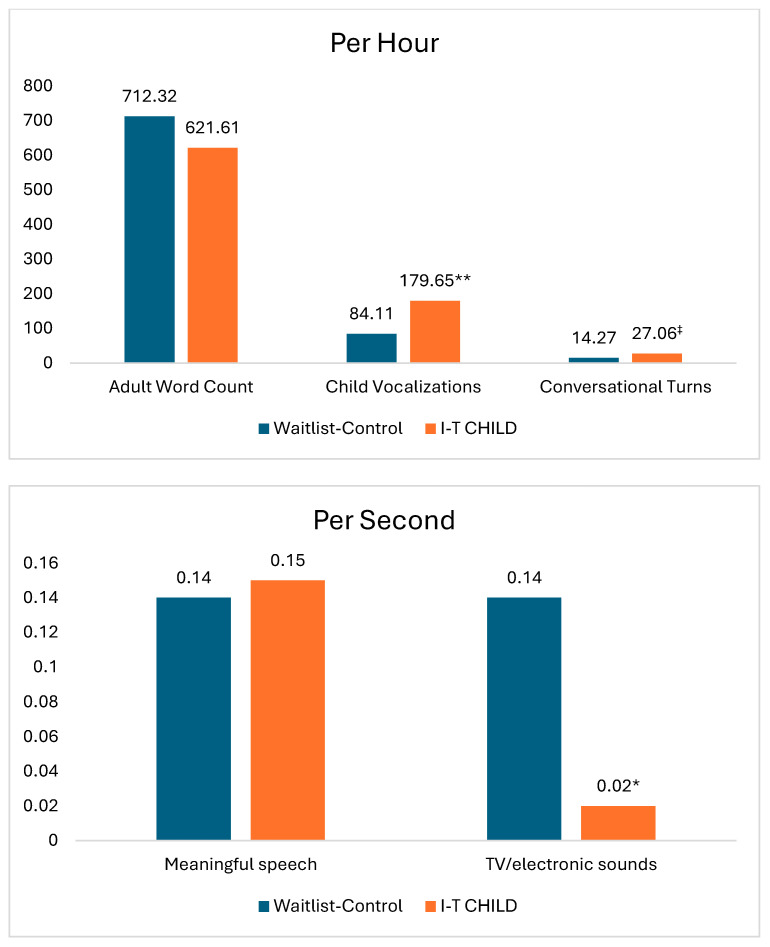
Adjusted mean posttest LENA scores based on HLM analyses. ^‡^ *p* < 0.10; * *p* < 0.05; ** *p* < 0.01.

As shown in Table 3 and Figure 1, HLM analyses controlling for pretest scores for each LENA variable revealed that no significant differences between I-T CHILD and waitlist-control groups were detected for adult word count (*t* = −0.47, *p* = 0.643, *ES* = −0.09) or meaningful speech (*t* = 0.27, *p* = 0.792, *ES* = 0.09). These findings suggest that the intervention was not associated with an overall increase in adult words directed to children or in the quantity of discernible speech sounds in the child’s environment. However, infants and toddlers in the I-T CHILD group produced significantly more child vocalizations than their peers in the waitlist-control group (*t* = 3.75, *p* = 0.002, *ES* = 1.50) and were also exposed to significantly less TV/electronic sound (*t* = −2.38, *p* = 0.032, *ES* = −0.97) than infants and toddlers in the waitlist-control condition. These findings suggest that providers coached using the I-T CHILD framework may have created more supportive and responsive environments, encouraging communicative attempts and minimizing noise interference in the background compared to providers in the waitlist-control condition. With respect to conversational turns, no statistically significant difference was observed (*t* = 1.95, *p* = 0.072); however, the moderately large effect size (*ES* = 0.65) suggests a trend indicating that I-T CHILD providers may have engaged in more contingent responding than the waitlist-control providers. This trend supports the possibility that I-T CHILD exposure increased providers’ responsiveness to children’s communicative bids, even if statistical significance was not reached in the current sample.

## 4. Discussion

This study seeks to examine the link between fostering a mentally healthy climate and the richness of the language environment in family child care programs. The study is timely as the intervention was delivered to a systemically disinvested population of essential workers at a time of heightened global (mental) health crises, the COVID-19 pandemic. Concerns over “learning loss” left many families with heightened levels of stress and mental health concerns [88]. These unprecedented levels of mental health challenges were likewise experienced by the ECCE workforce [89]. When states and cities were ordered to shut down their doors to curb the spread of the virus, many local leaders urged ECCE programs, especially family child care providers, to remain open for essential workers such as medical and food service personnel. Family child care providers—whether or not they were labeled formally as essential workers—kept the local economy functioning. During this difficult period when the mental health cost to society was high [90], ECMHC services delivered with the I-T CHILD framework came at an opportune moment in US history. The underlying premises of I-T CHILD are that (a) investing in high-quality early care and education yields greater benefits in later years [91] and (b) authentic, caring relationships are essential building blocks for healthy brain development [92,93]. 

### 4.1. Fostering a Climate of Mental Well-Being and Its Far-Reaching Effects Beyond SEL

Over a 12-week intervention period, consultant ratings of the mental health climate of family child care providers showed improvements across most dimensions. Of the dimensions that showed no statistical significance (adult awareness, adult affect, and child behaviors), they were already higher to begin with at pretest (i.e., close to what the CHILD-HBCC describes as “promoting” behaviors or close to a score of 1.0). The largest shift as indicated by the largest effect size is in adult–child interactions, with a notable shift from an average score of *baseline expectation* to *promoting*. According to the CHILD-HBCC manual, the adult–child interactions dimension examines (1) adult support and feedback provided to children, (2) provision of opportunities for child-centered activities, (3) facilitation of children’s verbal self-expression, (4) facilitation of children’s creative self-expression, and (5) provision of rich experiences (e.g., variety, stimulation, pace). The movement from *baseline expectation* to *promoting* suggests that instead of providing cursory support and feedback to children, providers lead the direction of activities with some input from children, allowing verbal and creative self-expression without active facilitation and providing activities that are more structured. Family child care providers after receiving I-T CHILD were providing more individualized support and feedback, facilitating children’s agency, facilitating a more language-rich environment, offering more opportunities for children’s creative pursuits, and offering more open-ended and loosely structured activities for children. One other notable shift was in the SEL dimension that moved from an average score of *undermining* to *baseline expectation*. Although SEL did not reach the promoting level, this shift means that prior to I-T CHILD, family child care providers were reacting negatively to children’s emotions (e.g., being dismissive, forbidding the expression of feelings), were reacting negatively when children were engaged in conflict with their peers, and were not facilitating positive peer relationships. After receiving I-T CHILD, family child care providers acknowledged children’s emotions, offered solutions to help children navigate conflict with their peers, and were acknowledging children’s positive peer interactions. These findings affirm the value of embedding SEL supports within coaching models targeting HBCC.

With respect to the main thesis of our study, our findings indicate that infants and toddlers in family child care programs assigned to the I-T CHILD intervention produce more vocalizations and are less exposed to electronic media sounds such as TV than infants and toddlers in the waitlist-control condition. Note that the calculated effect sizes for these language indicators are ~1.0–1.5 *SD* for a short intervention, about twice or more the effect sizes obtained for language and literacy outcomes in preschool studies (*ESs*~0.22–0.54) [94]. Although preliminary, this finding underscores the integral role of fostering a supportive climate for SEL in building healthy brain development unimpeded by the deleterious challenges poverty poses in these communities.

Although there were significantly more family child care providers in the intervention group that fell below the poverty threshold than there were in the waitlist-control group, this study has demonstrated that poverty is not a barrier to achieving these results. Indeed, we have demonstrated that I-T CHILD may maximize the longer-term cognitive potential of infants and toddlers in disinvested communities by harnessing the potential of providers’ caregiving interactions. 

### 4.2. Speaking with, Not Merely Talking to, Children

Although children in the I-T CHILD group exhibited significantly more vocalizations and were exposed to less background media noise than children in the waitlist-control group, adult word count and meaningful speech remained unchanged between groups. The trend we found toward more conversational turns, though not statistically significant, yields medium-to-large effect size (i.e., 0.65), suggesting greater contingent responding by providers, which is stressed in I-T CHILD. Contingent responding is not merely responding to children’s utterances. It is dependent on the previous verbalization of the child, and builds upon what the child has said, with the adult maintaining the flow of conversation based on the child’s continued contributions to the conversation—which can mean protoconversations with infants [51]. This is especially powerful because this RCT focused on infants and toddlers who verbalize or communicate in ways adults may not readily understand. This shift in conversational turns points to an environmental change that may support increased child talk potentially through increased emotional safety and reduced auditory interference. Prior studies examining the impact of teachers’ emotional support on children’s language outcomes have shown similar results [95]. Contrary to popular studies that focus on the contributions of adult words to child language [45], our study suggests that adult attunement to and affirmation of children’s linguistic attempts promotes a language-rich environment for these children. This is supported by Wang and others (2017) in their systematic review as they noted that, “In most cases, what matters is not the overall amount of adult talk, but the amount of adult–child conversational turns or parental verbal responsiveness” [82] (p. 309). A recent meta-analysis also found a small but consistent association between socioeconomic status and LENA-derived language metrics, suggesting that children from lower-income households tend to experience less enriched language environments [96]. However, our findings suggest that with intentional, culturally responsive consultation/coaching such as I-T CHILD, this pattern can be disrupted. Despite a higher proportion of family child care providers living below the poverty line in the intervention group, children in these settings demonstrated more vocalizations and reduced auditory interference, underscoring the transformative potential of emotionally supportive caregiving in disinvested communities. 

### 4.3. Serving the Underserved and Giving a Voice to the Voiceless

The COVID-19 pandemic exacerbated many of the preexisting inequities and challenges facing the ECCE industry. The intervention in this study offered mental health consultation services to home-based settings such as family child care, which are more representative of the US demographic landscape than center-based programs and serve a disproportionate number of racialized and working-class families who typically have less access to such resources [25,26]. Moreover, the combination of stigmatization of mental health and membership in a racially and ethnically minoritized group poses a double-edged sword that impedes treatment and promotion of their well-being [97]. Women of color, who are intentionally overrepresented in our sample of family child care providers, benefit from CHILD-informed consultation as evidenced in our RCT. 

Furthermore, the focus is on infants and toddlers who often are understudied in ECMHC research; they are given a voice through the use of LENA technology. LENA has been used in several research mostly focusing on the home environment [82] and less on the environment outside the home. Prior research tends to examine SEL and language development either through parent–child interactions or in relation to academic outcomes, particularly school readiness [73,98]. Our findings call for more contextualized approaches. The CHILD framework with the nine dimensions allows researchers to explore how multiple environmental and relational domains interact to shape children’s social-emotional and language outcomes. Avoiding a linear, static model of development allows for a richer understanding of children’s lived experiences in care environments other than the home. 

### 4.4. Implications on Equity: From Deficit to Opportunity

This study attempts to contribute to a body of evidence that will reduce inequalities in professional support for family child care providers [99]. It is also focused on facilitating the development of a supportive and healthy climate for learning and social interaction. The key outcome measure, language, is often neglected in favor of factors like teacher–child ratio, class size, and early learning stimulation experiences [76]. Given the documented limited access to these programs and the importance of accessing family child care settings especially for marginalized communities, this work attempts to provide experimentally-based evidence that serves their needs for social and emotional supports.

Our research team partnered closely with local community-based organizations that are critical for trust-building, culturally responsive recruitment, and retention—particularly in communities historically harmed by exploitative research practices. The majority of providers in our study preferred to communicate in a language other than English, emphasizing the importance of addressing linguistic racism and honoring providers’ preferred languages during training and research activities. Our work is one step closer in moving away from ideologies of deficits in investigations of development in racialized communities; we are one step closer to investigating the effects of programs that are culturally sustaining pedagogies and examine community assets. Our study seeks to elevate the critical role of family child care providers who often are judged as lower in delivering quality care and education compared to center-based providers—considering that they are assessed using Western lenses and methodologies.

### 4.5. Limitations and Future Directions 

This study is the first RCT of ECMHC delivered to family child care providers serving infants and toddlers in a racially and linguistically diverse minoritized urban community. While the current study included a diverse group or participants, the sample size was small partially due to recruitment challenges and attrition as a result of implementing the project at the height of the pandemic. Nevertheless, researchers need to recognize that for many marginalized communities, they are skeptical of research. Twelve of the fifty enrolled providers dropped out during the study, a reality that underscores the complex relationship many marginalized communities have with research. A larger sample size may have been needed to detect further effects of the intervention on children’s language outcomes. Intervention studies need to take the fraught relationship between marginalized communities with research in its design and allow for time to build trust and strengthen partnerships. Although this study incorporated a community-based liaison and multilingual protocols, future research must allow for more time to build trust and co-develop study goals with stakeholders. Moreover, more inclusive and equity-oriented methodologies must be applied. As Smith and others have noted, “research” can be viewed as extractive and harmful unless participatory approaches are used from the outset [100].

One other limitation in this study is the challenge in framing ECMHC as it is an esoteric concept. The different agencies that provided I-T CHILD also used other tools to work with their family child care providers. This ensures that ECMHC works organically with the specific needs of individual family child care providers, which may pose as a threat to internal validity. Using I-T CHILD as a unifying framework for consultation and conducting a randomized controlled trial, however, attenuates this concern. 

Finally, with respect to examining the language environment, while LENA is valid and reliable for capturing English, Spanish, Mandarin, and Cantonese, the preferred languages of providers, not all languages heard and spoken by the children have been validated (e.g., Fulani). Thus, there is a need for efforts to continue validating measures like LENA to better assess the quality of the language environment for multilingual learners who speak less common languages. In addition, even within validated languages, recent research has raised concerns about the accuracy of LENA’s automated estimates of conversational turns. Ramírez and others found that LENA significantly overestimated adult–child conversational turns compared to manual coding, particularly during infancy, with the discrepancies being largest at younger ages [101]. This finding highlights the importance of cautious interpretation and the need for complementary validation methods when using LENA to assess the quality of early language environments.

## 5. Conclusions

To conclude, ECMHC implemented with the I-T CHILD framework provides an invaluable service for family child care providers and the communities they serve. This study is the first to experimentally demonstrate the link between promoting a climate that supports mental health and the facilitation of a language-rich environment for infants and toddlers in family child care settings which can serve as a protective factor in challenging environments. One noteworthy aspect about the current study is that it is part of a much larger architecture that involves key policymakers, state agencies, child care resource and referral agencies providing ECMHC services, and a vast network of HBCC providers. The long-term goal is to establish sustainable statewide implementation of service delivery structures supporting infants and toddlers living in disinvested urban communities.

## Figures and Tables

**Table 1:** Guiding principles of the mental health climate.

	Undermining	Baseline Expectation	Promoting
**Pedagogy**	Harmful, detrimental	Perfunctory	Facilitative
	Highly regimented or laissez-faire	Somewhat inflexible or scripted	Flexible
	Adult-centered	Cursory incorporation of children’s interests	Child-centered
	Highly restrictive, dismissive, or overly permissive	Limiting	Empowering
	Reactive, neglectful, or disinterested	Routine or supervisory	Proactive, interested, and engaged
	Biased, intolerant, excluding, or disrespectful	Fair, equal, or respectful	Equitable, inclusive, and egalitarian
**Affect**	Disingenuous	Contrived or forced	Authentic
	Nonchalant or angry; antagonistic	Ostensibly nice	Warm and friendly
	Flat, ambiguous, negative, or mismatched	Superficially positive	Appropriately and responsively positive

**Table 2:** Adjusted LENA scores based on HLM analyses.

		Pretest					Posttest			
	Waitlist	I-T CHILD				Waitlist	I-T CHILD			
	*M* (*SD*)	*M* (*SD*)	Coeff (*SE*)	*95% CI*	*p* *ES*	*M* (*SD*)	*M* (*SD*)	Coeff (*SE*)	*95% CI*	*p* *ES*
AWC/h	1645.95 (1829.17)	960.20 (561.20)	−685.85 (567.710)	−1857.60–485.90	0.239 −0.52	712.32 (1298.51)	621.61 (373.38)	−90.71 (191.76)	−1857.60–485.90	0.643 −0.09
CV/h	134.93 (82.29)	138.51 (87.16)	3.58 (29.94)	−58.22–65.38	0.906 0.04	84.11 (47.55)	179.65 (83.02)	95.54 (25.49) **	−58.22–65.38	0.002 1.50
CT/h	29.27 (17.16)	25.57 (15.24)	−3.70 (5.98)	−16.04–8.64	0.542 −0.23	14.27 (21.40)	27.06 (16.41)	12.79 (6.57)	−16.04–8.64	0.072 0.65
TV/s	0.12 (0.16)	0.14 (0.15)	0.02 (0.05)	−0.08–0.12	0.253 0.15	0.14 (0.12)	0.02 (0.15)	0.13 (0.05) *	−0.08–0.12	0.032 −0.97
Speech/s	0.24 (0.10)	0.20 (0.06)	−0.04 (0.03)	−0.10–0.02	0.668 −0.47	0.14 (0.08)	0.15 (0.06)	0.01 (0.02)	−0.10–0.02	0.792 0.09

AWC = adult word count; CV = child vocalizations; CT = conversational turns; TV = electronic media exposure; Speech = meaningful speech. * *p* < 0.05; ** *p* < 0.01.

**Table 3:** Consultant ratings of mental health climate.

CHILD-HBCC Dimensions	Pretest	Posttest	*t*	*ES*
Transitions	0.30	0.79	2.89 *	0.67
Directions and Rules	0.14	0.82	3.69 **	0.85
Social and Emotional Learning	−0.28	0.39	4.11 ***	0.95
Adult Awareness	0.77	1.11	1.91	0.44
Adult Affect	1.06	1.20	0.74	0.17
Adult Cooperation	0.82	1.34	2.66 *	0.67
Adult–Child Interactions	0.43	1.08	5.01 ***	1.16
Individualized and Developmentally Appropriate Practices	0.02	0.77	3.96 **	0.91
Child Behaviors	0.93	1.20	1.56	0.36

* *p* < 0.05; ** *p* < 0.01; *** *p* < 0.001.

## Data Availability

The data used are confidential to protect the sensitive circumstances surrounding the study participants. Data are available on request from the corresponding author.
